# Regulation and Function of TMEM16F in Renal Podocytes

**DOI:** 10.3390/ijms19061798

**Published:** 2018-06-18

**Authors:** Laura K. Schenk, Jiraporn Ousingsawat, Boris V. Skryabin, Rainer Schreiber, Hermann Pavenstädt, Karl Kunzelmann

**Affiliations:** 1 Department of Nephrology, Hypertension and Rheumatology, University Hospital Muenster, 48149 Muenster, Germany; laura-katharina.schenk@charite.de (L.K.S.); hermann.pavenstaedt@ukmuenster.de (H.P.); 2 Institut für Physiologie, Universität Regensburg, Universitätsstraße 31, D-93053 Regensburg, Germany; jiraporn.ousingsawat@vkl.uni-regensburg.de (J.O.); rainer.schreiber@vkl.uni-regensburg.de (R.S.); 3 Transgenic Animal and Genetic Engineering Models (TRAM), Department of Medicine, Westfälischen, Wilhelms–Universität Münster, 48149 Münster, Germany; skryabi@uni-muenster.de

**Keywords:** TMEM16F, anoctamin 6, kidney, renal ion channels, chloride channel

## Abstract

The Ca^2+^-activated phospholipid scramblase and ion channel TMEM16F is expressed in podocytes of renal glomeruli. Podocytes are specialized cells that form interdigitating foot processes as an essential component of the glomerular filter. These cells, which participate in generation of the primary urine, are often affected during primary glomerular diseases, such as glomerulonephritis and secondary hypertensive or diabetic nephropathy, which always leads to proteinuria. Because the function of podocytes is known to be controlled by intracellular Ca^2+^ signaling, it is important to know about the role of Ca^2+^-activated TMEM16F in these cells. To that end, we generated an inducible TMEM16F knockdown in the podocyte cell line AB8, and produced a conditional mouse model with knockout of TMEM16F in podocytes and renal epithelial cells of the nephron. We found that knockdown of TMEM16F did not produce proteinuria or any obvious phenotypic changes. Knockdown of TMEM16F affected cell death of tubular epithelial cells but not of glomerular podocytes when analyzed in TUNEL assays. Surprisingly, and in contrast to other cell types, TMEM16F did not control intracellular Ca^2+^ signaling and was not responsible for Ca^2+^-activated whole cell currents in podocytes. TMEM16F levels in podocytes were enhanced after inhibition of the endolysosomal pathway and after treatment with angiotensin II. Renal knockout of TMEM16F did not compromise renal morphology and serum electrolytes. Taken together, in contrast to other cell types, such as platelets, bone cells, and immune cells, TMEM16F shows little effect on basal properties of podocytes and does not appear to be essential for renal function.

## 1. Introduction

The family of TMEM16 proteins (anoctamins) consists of a completely novel type of Ca^2+^-regulated ion channel/phospholipid transporter with unusual structures [1,2]. These channels/transporters are abundant with high expression in the kidney [3,4]. Filtration and generation of primary urine takes place in renal glomeruli, which contain podocytes, a unique cell type with interdigitating foot processes that form a glomerular filter [5]. Primary glomerular diseases, such as glomerulonephritis and secondary renal damage during hypertensive or diabetic nephropathy, frequently result in damage of the glomerular filtration barrier.

Ca^2+^-dependent intracellular signaling plays a key role in podocyte damage. Thus, gain-of-function mutations in the calcium channel TRPC6 cause focal-segmental glomerulosclerosis. Besides inherited defects in Ca^2+^-regulated proteins, imbalance in hormones regulating intracellular calcium, such as angiotensin or insulin, can have harmful effects [6,7]. Angiotensin II (Ang II) increases intracellular Ca^2+^ in podocytes via the angiotensin II receptor 1 (AT1R) [7]. Chronic overactivation of the renin-angiotensin-aldosterone signaling cascade is associated with renal failure, which can be decelerated by inhibitors of the hormonal cascade [8,9]. It is therefore important to understand the function and regulation of Ca^2+^-dependent TMEM16F in podocytes.

TMEM16F belongs to a family of 10 Ca^2+^-regulated proteins (TMEM16A-K, anoctamin 1–10) [10]. TMEM16F is broadly expressed in many tissues, including kidney epithelial cells. TMEM16F is a Ca^2+^-activated phospholipid scramblase that moves phospholipids from the inner to the outer leaflet of the plasma membrane and vice versa. Thus, it exposes phosphatidylserine in the outer membrane leaflet that leads to structural changes during activation of platelets, participates in membrane blebbing and cell death, and represents an immunological eat-me signal detected by binding of annexin V [10,11,12]. Thus, TMEM16F and proteins of the Xk-related family, specifically Xkr8, counteract the asymmetric bilayer composition via passive, non-directional transport [13]. The large twin pores within the TMEM16F dimer that conduct phospholipid head groups seem to also allow the permeation of ions. Therefore, activation of TMEM16F is paralleled by Cl^−^ and cation currents depending on the level of activation [12,14,15,16].

The lipid composition of the outer plasma membrane may change during cell death or in response to cellular stimulation or paracrine signaling. Thus, TMEM16F induces phospholipid scrambling during apoptosis or ATP-induced cell activation [12,17]. We showed previously that knockdown of TMEM16F modifies phosphatidylserine exposure and cell survival in HEK293 cells and found regulation of intracellular kinases, such as AKT-dependent signaling, by TMEM16F [18]. A conventional TMEM16F knockout mouse has been studied earlier in detail and shows defects in bone mineralization and defects in macrophages [12]. The Ca^2+^-regulated TMEM16F scramblase and ion channel is also expressed in human and murine kidneys. Given the relevance of intracellular Ca^2+^ signaling and Ca^2+^-regulated chloride currents in podocytes, we analyzed the regulation and function of TMEM16F in this cell type. To this end, we established an inducible TMEM16F knockdown in a human podocyte cell line and generated a renal epithelial specific TMEM16F knockout mouse. Compared with the renal epithelial specific knockout of TMEM16A, which induced a mild renal phenotype, knockout of TMEM16F did not show a renal defect or any obvious phenotypic changes [3]. Knockout of TMEM16F in podocytes had no effect on Ca^2+^-activated Cl^−^ currents or Ca^2+^ signaling, and only slightly affected cell death when compared with other cell types [12].

## 2. Results

### 2.1. TMEM16F is Expressed in Podocytes of Human and Murine Glomeruli

**Figure 1:**
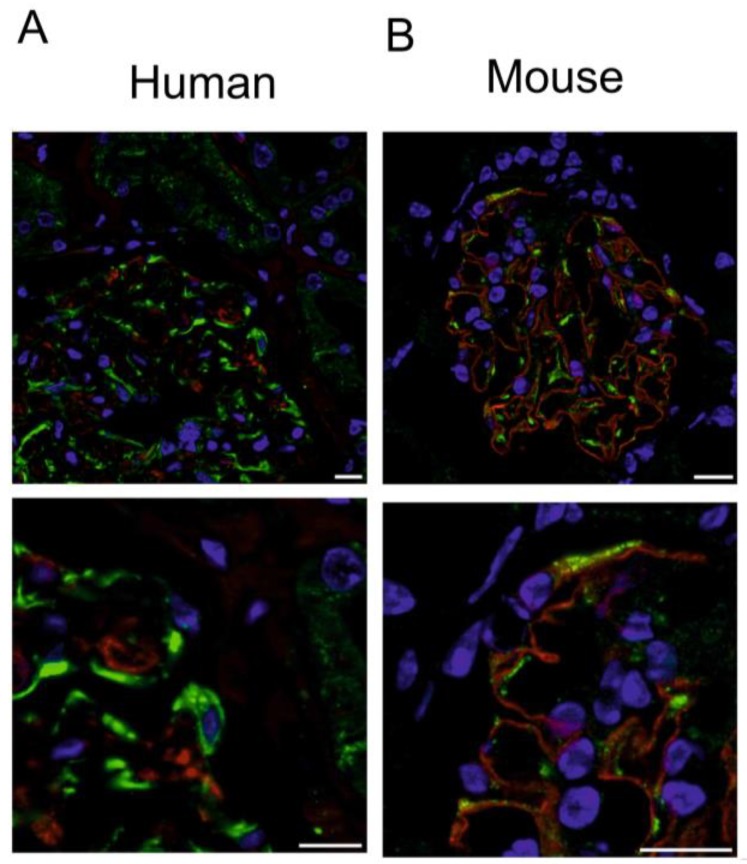
Expression of TMEM16F in human and mouse glomeruli. (**A**) Immunohistochemistry of cortical sections of an adult human kidney showing TMEM16F (green) and the podocyte marker nephrin (red). Cell nuclei are marked by DAPI (blue). Podocytes seem to express TMEM16F in plasma membrane and cytoplasm. Scale bar 10 µm. (**B**) Immunohistochemistry of a cortical kidney section of an adult mouse showing TMEM16F (green) and the podocyte marker nephrin (red). TMEM16F does not colocalize with nephrin in foot processes but is rather localized in the cell body of podocytes.

**Figure 2:**
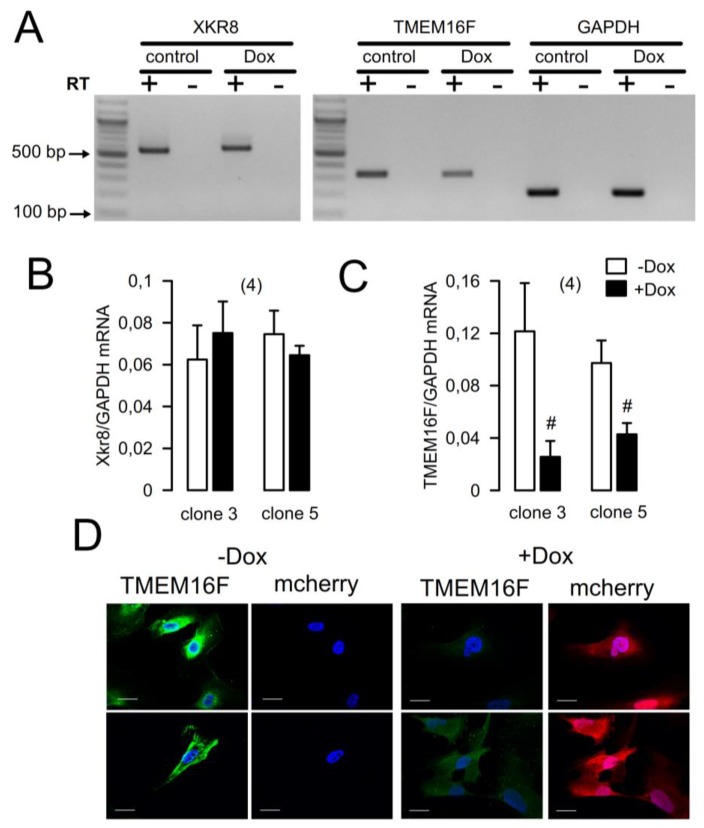
Expression of TMEM16F and Xkr8 in AB8 podocytes. (**A**) Analysis of expression of Xkr8 and TMEM16F by RT-PCR in AB8 podocytes expressing TMEM16F-shRNA3 or shRNA5. (**B**,**C**) Summary of the expression of Xkr8 and TMEM16F in the absence or presence of doxycycline (doxy; 125 ng/mL). (**D**) Immunofluorescence of TMEM16F and Cherry fluorescence in AB8 cells in the absence and presence of dox. Mean ± standard error of the mean (SEM) (number of experiments). # significant difference when compared to -Dox (*p* < 0.05; unpaired Student’s *t* test).

Immunofluorescence demonstrates expression of TMEM16F in human and mouse glomeruli (Figure 1). Costaining of TMEM16F and nephrin, a typical protein of the glomerular slit diaphragm, indicates coexpression in glomerular podocytes. TMEM16F was not found to be colocalized with nephrin in foot processes, which may correspond to the absence of changes in glomerular filtration and the absence of proteinuria in these animals. The present results are similar to the results of a recent study with the close paralogue TMEM16A. TMEM16A is also not colocalized with nephrin in the foot process [3]. TMEM16F was localized both in the plasma membrane and the cytoplasm of podocytes (also compare Figure 2D). Similar patterns of TMEM16F expression were detected in human and mouse glomeruli.

### 2.2. Inducible Knockdown of TMEM16F in AB8 Human Podocytes

**Figure 3:**
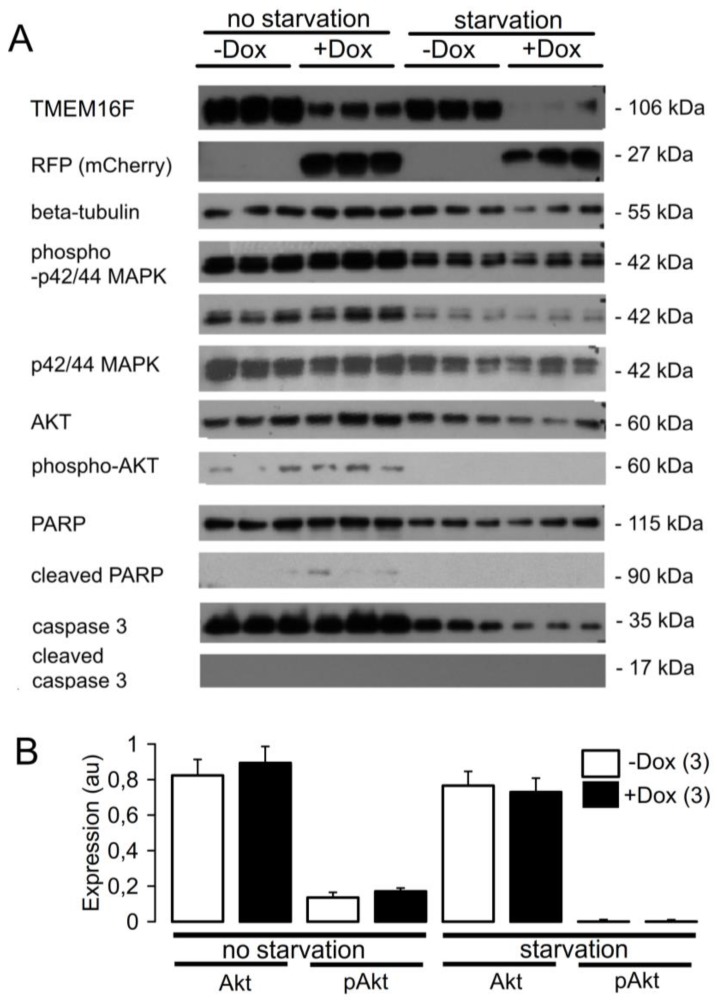
Knockdown of TMEM16F in podocytes had little effects on the expression of proteins related to cell proliferation/cell cycle or cell death. (**A**) Western blot analyses of three independent lysates from AB8 shTMEM16F-3 cells. Samples that have been induced with Doxycycline for 7 days are indicated as TMEM16F KD. Cells for the samples shown in the left panel were cultured under standard conditions, and cells for control and knockdown samples shown on the right were starved in serum-free medium for 48 h prior to harvesting. Equal loading, efficient induction, and knockdown of the target protein TMEM16F were verified by immunoblotting for TMEM16F, red fluorescent protein (RFP) cassette (mCherry), and beta tubulin. Western blots were performed for p42/44 MAPK, Akt, phospho-p42/44 MAPK (at a long (upper blot) and a short (lower blot) exposure time), phospho-Akt, and indicator proteins of apoptosis (cleavage of Caspase 3 and poly-ADP-Ribose-Polymerase (PARP)). (**B**) Densitometry analysis of expression of Akt and phospho-Akt relative to ß-tubulin (arbitrary units, au). Apart from decreased phosphorylation of AKT at T308 in starved cells, there were no quantitative differences in signaling proteins included in this screen. Mean ± SEM (number of experiments).

Functional analyses of naïve podocytes are notoriously difficult. In order to examine the functional role of TMEM16F in podocytes, we therefore generated an inducible TMEM16F-knockout cell line from the immortalized human podocyte cell line AB8/13 [19]. AB8 cells express the podocyte-specific cytoskeletal proteins nephrin and synaptopodin and form filopodia and lamellipodia. For knockdown of TMEM16F, an inducible knockdown (KD) was generated employing pInducer10 vector. Five short hairpin RNA sequences were screened with regard to efficient reduction of TMEM16F expression. The short hairpin RNAs shTMEM16F-3 (clone 3) and shTMEM16F-5 (clone 5) reduced expression of TMEM16F mRNA significantly (Figure 2A,C). Expression of TMEM16A was not detected in AB8 cells (data not shown) and expression of the TMEM16F-independent phospholipid scramblase Xkr8 was not affected by shRNA (Figure 2A,B). shTMEM16F-3 and shTMEM16F-5 largely reduced expression of TMEM16F protein (Figure 2A,C). A doxycycline-induction for 3–5 days was sufficient to decrease significantly TMEM16F expression, but for the assessment of steady state effects of TMEM16F knockdown on cellular pathways, we chose an induction period of 7 days. This led to a marked decrease in TMEM16F protein. Induction was also verified by the expression of the RFP (mCherry) cassette (Figure 3A).

### 2.3. Knockdown of TMEM16F Did Not Affect Expression of Proteins Related to Cell Cycle or Cell Proliferation

We have previously shown that knockdown of TMEM16F decreased the viability of HEK293 cells [18]. Reduced viability was paralleled by enhanced phosphorylation of the serine/threonine-specific protein kinase B, also known as Akt, at the activating T308-site, as well as other pro-proliferative signaling pathways, including cyclin D1. Phosphorylation of p42/44 MAPK and proteins of the mTOR pathway, however, remained unaltered in HEK293 cells. Here, we examined the role of TMEM16F for expression of proteins related to pro-proliferative and pro-apoptotic pathways, but detected no obvious changes upon knockdown of TMEM16F (Figure 3). TMEM16F knockdown did not affect p42/44 MAPK phosphorylation, cleavage of pro-apoptotic caspase 3, or cleavage of poly-ADP-Ribose-Polymerase (PARP). Also, after starvation of the cells for 48 h, no effect was observed by TMEM16F knockdown on the screened intracellular pathways (Figure 3, right lanes). Finally, MTT assays did not indicate any change in cell proliferation or viability upon knockdown of TMEM16F (data not shown).

### 2.4. Knockdown of TMEM16F Affects Cell Death in Tubular Epithelial Cells but not in Glomerular Podocytes

**Figure 4:**
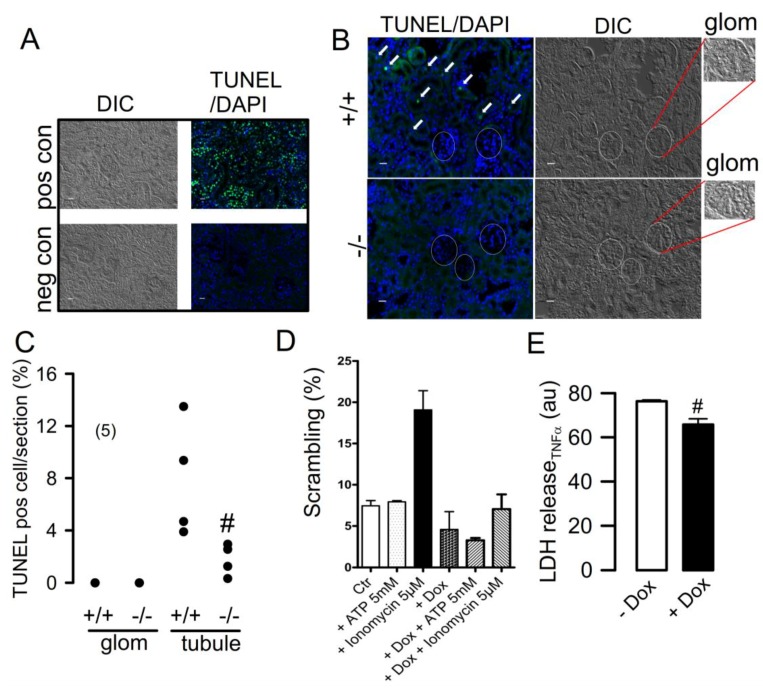
Cell death in renal epithelial cells. (**A**) Positive control (+DNAse I) and negative control (-rTerminal Deoxynucleotidyl Transferase) for TUNEL assays. (**B**,**C**) TUNEL staining in TMEM16F+/+ and TMEM16F−/− kidneys. No signals were detected in glomerula of both +/+ and −/− kidneys and only very few TUNEL-positive cells were seen in tubules of −/− kidneys. # significant difference when compared to +/+ (*p* <0.05; unpaired Student’s *t* test). White circles shown in B indicate localization of glomeruli, which are also shown as enhanced magnified insets (glom). (**D**) Phospholipid scrambling (annexinV positivity) upon stimulation with ATP (5 mM) or the Ca^2+^ ionophore ionomycin (5 µM) in AB8-TMEM16F+/+ (-dox) and AB8-TMEM16F−/− (+dox) podocytes. (**E**) LDH (lactate dehydrogenase) release induced by TNFα in AB8-TMEM16F+/+ (−dox) and AB8-TMEM16F−/− (+dox) podocytes. Mean ± SEM (number of experiments). ^#^ significant difference when compared to −Dox (*p* < 0.05; unpaired Student’s *t* test).

In contrast to other cell types, proliferation of podocytes was not affected by knockdown of TMEM16F [18,20,21,22]. We performed TUNEL stainings in renal sections from wild-type (wt) and TMEM16F knockout animals after performing control experiments in wild-type tissue Figure 4A). Remarkably, no TUNEL-positive cells were detected in either wt or TMEM16F-KO podocytes (encircled areas and insets (glom) in Figure 4B. In contrast, TUNEL-positive tubular epithelial cells were detected in wild-type kidneys, which were significantly reduced in kidneys from TMEM16F−/− cells (Figure 4B,C). Phospholipid scrambling was induced by ionomycin in AB8-TMEM16F+/+ cells, but was missing in AB8-TMEM16F−/− cells (Figure 4D). Finally, TNFα-induced cell death (as measured by LDH release) was only slightly compromised in AB8-TMEM16F−/− cells. Thus, a clear role of TMEM16F for cell death could not be detected in podocytes, which appear to have generally a lower apoptotic activity.

### 2.5. TMEM16F Does not Control Intracellular Ca^2+^ Signaling and Is not Responsible for Ca^2+^-Activated Whole-Cell Currents in Podocytes

**Figure 5:**
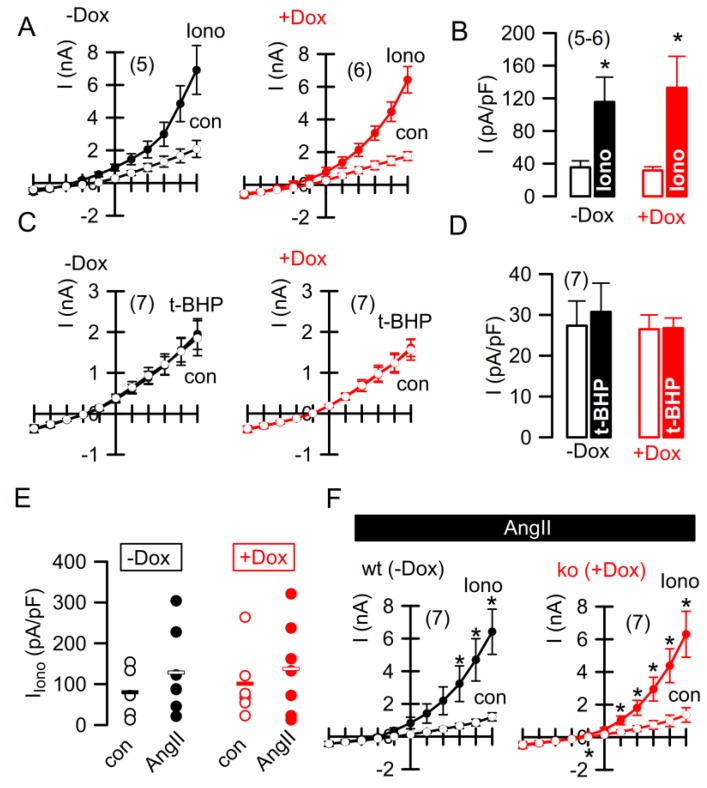
Ca^2+^-activated whole-cell currents in AB8 podocytes. (**A**,**B**) Current voltage relationships of whole-cell ion currents and current densities activated by the Ca^2+^ ionophore ionomycin (1 µM) in AB8 podocytes in the presence (−Dox) or absence (+Dox) of TMEM16F. (**C**,**D**) Current voltage relationships of whole-cell ion currents and current densities in the absence (con) or presence of the ROS donor and lipid peroxydizer tert-butyl hydroperoxide (tBHP;100 µM/2 h). (**E**,**F**) Summary of ionomycin-induced whole-cell currents and current/voltage relationships in AB8-TMEM16F+/+ (−dox) and AB8-TMEM16F−/− (+dox) podocytes and effect of 48 h incubation with angiotensin II (AngII) (1 µM). Mean ± SEM (number of experiments). * significant effect of ionomycin (*p* < 0.05; paired Student’s *t* test).

We examined other cellular parameters that have been demonstrated earlier to be affected by expression of TMEM16F. Phospholipid scrambling and whole-cell currents are induced by expression and activation of TMEM16F [12,15]. Moreover, TMEM16F augments agonist-induced intracellular Ca^2+^ signals by supporting Ca^2+^ influx [23,24,25]. We compared whole-cell currents and Ca^2+^ signaling in podocytes in the presence (−doxycyclin; −DOX) or absence (+doxycycline; +DOX) of TMEM16F. In all experiments, the experimental bath was continuously perfused (200 mL bath volume, perfusion rate 8 mL/min) with a bicarbonate-free Ringer solution in order to reduce possible contributions of additional bicarbonate-transporting membrane proteins, such as those of the SLC26A family. As the bath solution is continuously exchanged, extracellular pH changes cannot occur. The pH was 7.4 throughout the experiment and was measured using a micro-pH meter. In the whole-cell patch clamp experiments, an ion current was activated by an increase of intracellular Ca^2+^ with ionomycin (Iono, 1 µM). Surprisingly, activation of the whole-cell current was completely independent of TMEM16F-knockdown (+DOX), which was different to the results of many previous studies [12,16,26] (Figure 5A,B). Moreover, tert-butyl hydroperoxide (tBHP;100 µM/2 h), which has been shown previously to activate TMEM16F whole-cell currents, did not activate any ion currents independent of TMEM16F knockdown by doxycycline [16,27] (Figure 5C,D). Finally, pre-incubation with Angitensin II (AngII), which upregulates expression of TMEM16F, did not induce an TMEM16F-dependent, Ca^2+^-activated whole-cell current (Figure 5E,F). Thus, it is very likely that ionomycin activates another TMEM16 channel expressed in mouse podocytes. As there are many paralogues expressed in mouse glomeruli (ANO 1,3,5,6,8,9), it is possible that another anoctamin (not TMEM16A or TMEM16F) is in charge of the ionomycin-activated Cl^−^ current. This is rather likely as niclosamide, a very powerful inhibitor of TMEM16 proteins (Miner et al., 2017), inhibited ionomycin-activated whole-cell currents (5.34 ± 0,84 nA; Iono; *n* = 4) versus 3.1 ± 0,72 nA; Iono/niclosamide; *n* = 4).

**Figure 6:**
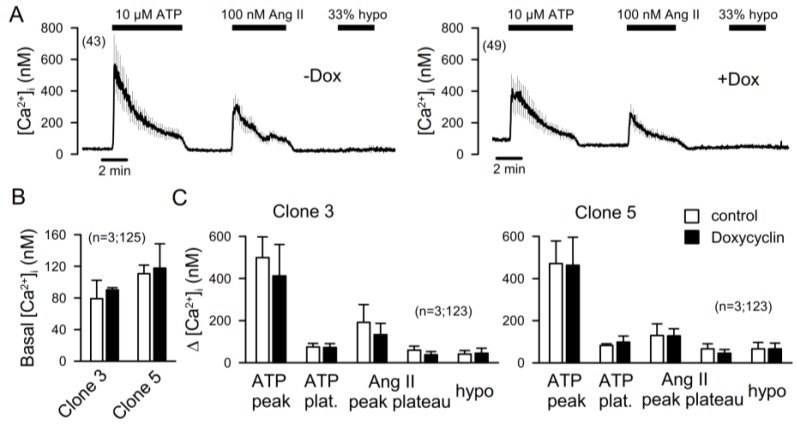
Ca^2+^ signaling in AB8 podocytes. (**A**,**B**) Increase in intracellular Ca^2+^ ([Ca^2+^]_i_) by stimulation with ATP (10 µM), Ang II (100 nM), or hypotonic bath solution (33%) in the presence (−Dox) or absence (+Dox) of TMEM16F (summary curves). (**B**) Summary of basal Ca^2+^ levels in two different AB8 clones, IONO in the presence (−Dox) or absence (+Dox) of TMEM16F. (**C**) Summary of the effects of ATP, Ang II, and hypo on two AB8 clones in the presence (−Dox) or absence (+Dox) of TMEM16F. Mean ± SEM (number of experiments).

Intracellular Ca^2+^ levels were determined using Ca^2+^-dependent Fura2 fluorescence. To that end, cells were loaded with Fura2 prior to the experiment and basal Ca^2+^ levels were measured. Afterwards, cells were stimulated with agonists known to increase intracellular Ca^2+^, such as ATP (10 µM), angiotensin II (AngII; 100 nM), or hypotonic bath solution (33% hypo) [21]. ATP and AngII, but not 33% hypo, increased intracellular Ca^2+^ and demonstrated the typical peak (Ca^2+^ release from endoplasmic reticulum) and plateau (store-operated Ca^2+^ influx) increase. Rather surprising was that Ca^2+^ increase was entirely independent of TMEM16F expression as demonstrated in two independent podocyte clones (Figure 6).

### 2.6. Regulation of TMEM16F in Podocytes

**Figure 7:**
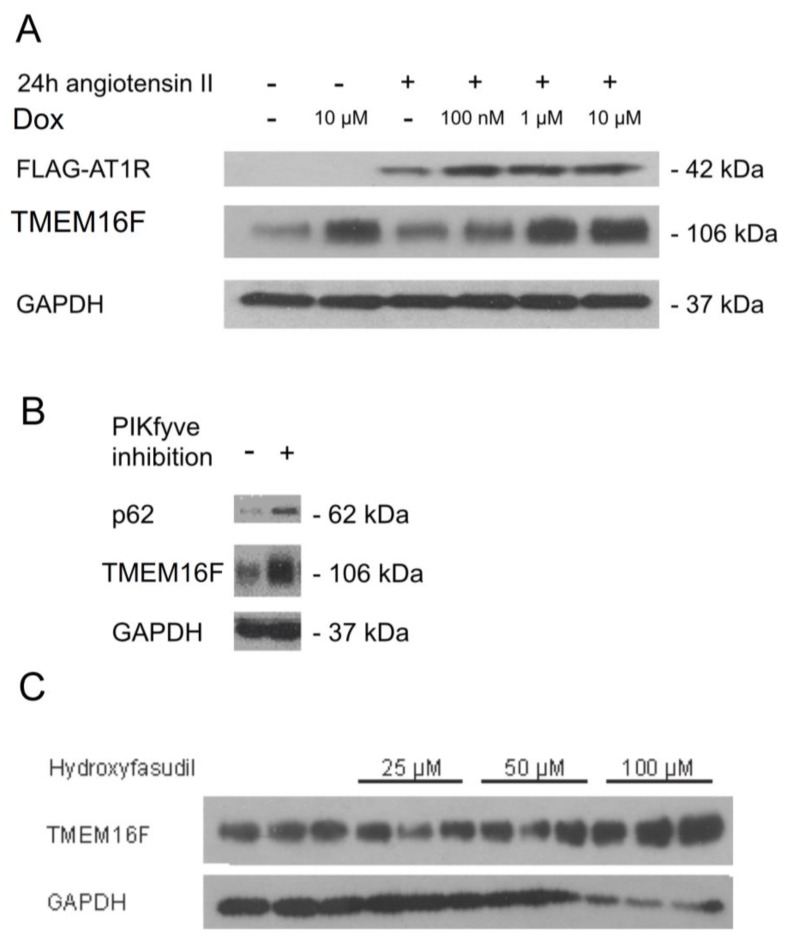
Regulation of TMEM16F in podocytes. (**A**) Western blot analysis indicating low endogenous non-detectable AT1R levels that were enhanced by incubation with Ang II and additional treatment with doxycycline (Dox). Ang II and additional expression of AT1R strongly augmented expression of TMEM16F. (**B**) Western blotting indicating cellular accumulation of nuclear pore glycoprotein 62 (p62) and TMEM16F during inhibition of PIKfyve. (**C**) Western blots indicating accumulation of TMEM16F in podocytes by treatment with the inhibitor of Rho kinase (ROCK), hydroxyfasudil.

Our data demonstrate that many of the TMEM16F-related cellular effects observed in freshly isolated tissues or cultured cells are not observed in podocytes, a finding that remains currently unexplained. We further examined whether pathological conditions are induced by altered TMEM16F expression in podocytes and whether knockout of TMEM16F causes a renal phenotype. Glomerular hemodynamics are substantially regulated by Ang II, and Ang II is known to affect the functional integrity of podocytes [5,6,8,28]. TMEM16F may contribute to Ang II-induced effects on activation and survival of podocytes. Along this line, we examined possible changes in TMEM16F expression during exposure to Ang II. We used AB8 cells with inducible expression of FLAG-tagged human AT1 receptors (AB8 3F-hAT1R) [29]. Low endogenous non-detectable AT1R levels were enhanced by incubation with Ang II and additional treatment with doxycycline (Figure 7A). Moreover, both Ang II and additional expression of AT1R strongly augmented expression of TMEM16F, which may suggest a functional relevance of TMEM16F for Ang II-related renal disease (Figure 7A).

As some membrane proteins are regulated by endolysosomal recycling, we examined whether inhibition of 1-phosphatidylinositol 3-phosphate 5-kinase (PIKfyve) would affect expression of TMEME16F in AB8 podoctyes. PIKfyve is an essential enzyme of the endolysosomal pathway, which was inhibited by YM201636 (800 nM, 5 h). Indeed, inhibition of PIKfyve led to an accumulation of TMEM16F protein in podocytes (Figure 7B). Parallel accumulation of the nuclear pore glycoprotein 62 (p62) was used as a positive control. Finally, the inhibitor of Rho kinase (ROCK), hydroxyfasudil, also led to an accumulation of TMEM16F in podocytes (Figure 7C). Taken together, TMEM16F is regulated by endolysosomal recycling in human podocytes.

### 2.7. Knockout of TMEM16F in Podocytes Does Not Compromise Renal Morphology and Renal Function

**Figure 8:**
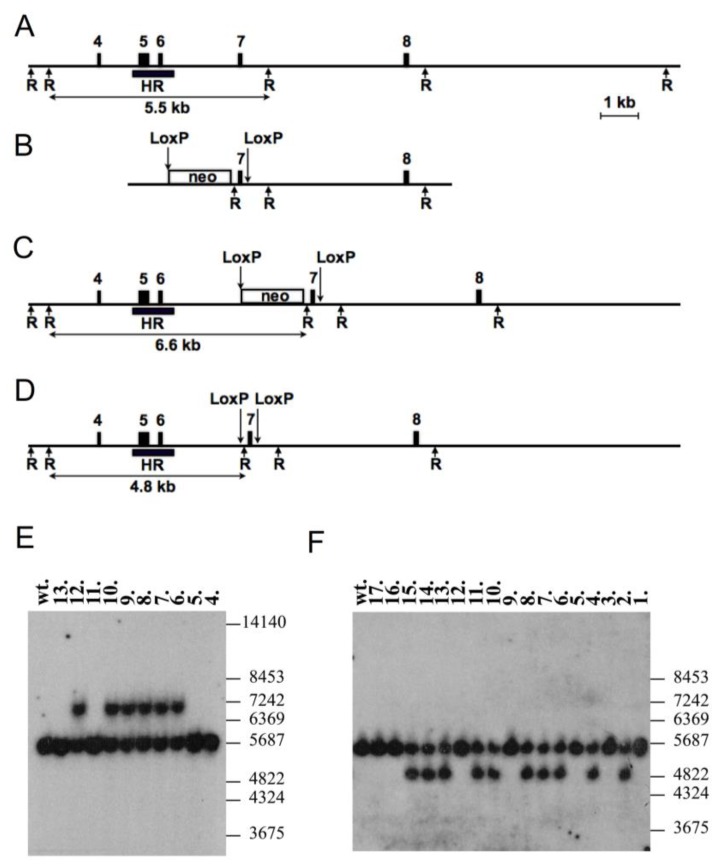
Generation of TMEM16F floxed mice. (**A**–**D**) Targeting of mouse TMEM16F gene, exon 7. The intronic and intergenic regions are presented as a line, and exons are displayed as filled boxes with numeration. The neomycin resistance cassette is marked (neo) and flanked by the FRT sites (not shown). The arrows above correspond to the LoxP sequences, and the arrows below correspond to restriction endonuclease sites *Eco*RI (R). The black box corresponds to the Southern probe sequence (HR). The expected sizes of restriction DNA fragments are labeled below in kb. (**A**) Wild-type (wt) locus. (**B**) Targeted vector structure (without negative selection marker and plasmid backbone). (**C**) Mouse genomic locus after the homologous recombination. The “neo” cassette is present in intron 6, and exon 7 flanked by two LoxP sites. (**D**) Deletion of the “neo” cassette after crossing with the FLPe-deleter mice. The exon 7 is flanked by two LoxP sites. (**E**) Southern blot analysis of DNAs isolated from F1 mouse tail biopsy (4–13) and hybridized with the HR probe. With the help of the *Eco*RI enzymatic digestion, we detected wt allele 5.5 kb and targeted allele 6.6 kb. DNA samples 6–10 and 12 contain correctly targeted *TMEM16F* gene. Positions of the size marker (in bp) are shown on the right. (**F**) Southern blot analysis of DNAs isolated from mouse tail biopsy (1–17) of F2 offspring after crossing of the *TMEM16F^+/-^* with the FLPe-expressing transgenic mice. *Eco*RI enzymatic digestion detected animals 2, 4, 6–9, 10, 11, and 13–15 with the deleted “neo” cassette.

**Figure 9:**
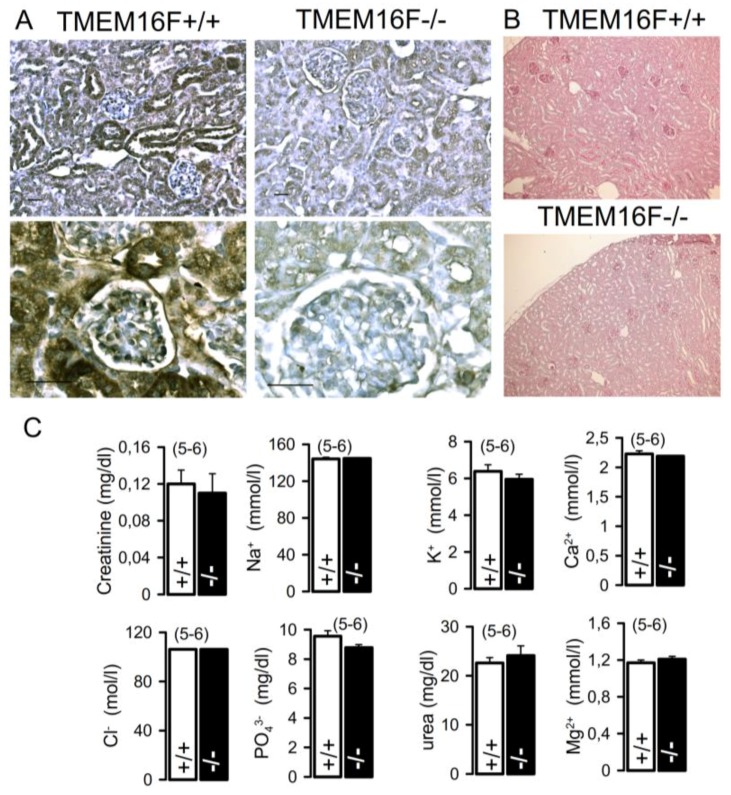
Lack of a renal phenotype by renal TMEM16F knockout. (**A**) Immunohistochemistry of TMEM16F(flox/flox) Six2Cre mice. Cre-negative animals, which were used as a littermate control in our study, display a normal expression of TMEM16F. Cre-positive animals (TMEM16F−/−) lack expression of TMEM16F in the kidney parenchyma. (**B**) Periodic acid Schiff stain of kidney sections of Cre-negative (control; TMEM16F+/+) and Cre-positive (TMEM16F−/−) animals. The parenchyma appears normal and cysts are absent in the TMEM16F−/− kidney. (**C**) Summaries for serum creatinine, urea nitrogen, and serum electrolyte concentrations, which did not differ between TMEM16F+/+ and TMEM16F−/− animals. Mean ± SEM (number of experiments).

The present data suggest that expression of TMEM16F in renal podocytes has little impact on podocyte function and, in contrast to other cell types, does not affect cellular parameters, such as Ca^2+^ signaling, Ca^2+^-activated ion currents, or cell death. In order to determine whether TMEM16F is important for renal function, we generated a renal TMEM16F knockout mouse model (Figure 8; c.f. Methods). Mice with the loxP-flanked TMEM16F gene, TMEM16F(lox/lox) mice, were bred with SIX2 Cre transgenic mice [30]. SIX2 expression starts early during nephrogenesis; consequently, the progeny lack expression of TMEM16F from the cap mesenchymal stage in all descendants that form the renal nephron [30]. Crossbreeding of mice with a floxed TMEM16F allele with SIX2 Cre mice led to a constitutive knockout of TMEM16F in glomerular podocytes and tubular epithelial cells while Cre-negative littermate controls displayed normal expression of TMEM16F (Figure 9A). Renal histology of TMEM16F−/− mice confirmed an intact structure, i.e., corticomedullary differentiation was intact, glomeruli and tubuli appeared normal, and renal cysts were not observed (Figure 9B). Renal TMEM16F knockout mice grew like their wt littermates, and none of the TMEM16F−/− animals developed albuminuria or gross proteinuria. The serum creatinine, urea nitrogen, and serum electrolyte concentrations did not differ between TMEM16F+/+ and TMEM16F−/− animals (Figure 9C). The data suggest that TMEM16F is not essential to maintain the selectivity of the podocyte slit membrane.

## 3. Discussion

TMEM16F is a Ca^2+^-activated phospholipid scramblase and generates whole-cell currents when activated by an increase in intracellular Ca^2+^ [10,12,15]. TMEM16F has essential functions in platelets, bone cells, macrophages, and T-lymphocytes [15,26,31,32,33,34,35]. It is essential for blood coagulation, bone mineralization, immune defense, and protein shedding. TMEM16F also fulfills basal cellular functions during Ca^2+^ signaling, volume regulation, blebbing of the plasma membrane, and cell death [12,21,23,24,27,36]. It was therefore interesting to examine a possible role of TMEM16F in renal epithelial cells and in particular in renal glomerular podocytes.

Rather surprisingly, knockout of TMEM16F did not compromise renal morphology and does not seem to be essential for maintaining the selectivity of the podocyte slit membrane. Thus, TMEM16F-knockout mice did not develop any obvious proteinuria. The present data do not provide any evidence for a role of TMEM16F in regulation of glomerular filtration, i.e., protein filtration. As knockout of the Ca^2+^-activated Cl^−^ channel TMEM16A did not induce proteinuria or any obvious podocyte defect in an earlier study [3], we hoped to identify the common paralogue TMEM16F as the essential Ca^2+^-activated Cl^−^ channel in podocytes. The fact that Ca^2+^-activated Cl^−^ currents are present in podocytes independent of TMEM16F suggests a role of another TMEM16 protein as expression of CFTR was not detected. It would be interesting to examine in future studies whether LRRC8A plays a role for Ca^2+^-activated whole-cell currents in renal podocytes.

Moreover, changes in cellular properties typically related to knockdown of TMEM16F were not observed in podocytes, albeit knockdown of TMEM16F blocked Ca^2+^-activated phospholipid scrambling (Figure 4D). Thus, TMEM16F does not seem to have a major role in podocytes. Although knockout of TMEM16F did not produce proteinuria under basal conditions, TMEM16F could still be relevant during pathological conditions. In fact, some of the present data suggest that TMEM16F may contribute to Ang II-induced activation and survival of podocytes. Glomerular hemodynamics are substantially regulated by Ang II, and Ang II is known to affect the functional integrity of podocytes [5,6,8,28]. TMEM16F may contribute to the Ang II-induced effects on activation and survival of podocytes. At any rate, the rate of spontaneous apoptotic cell death appears to be very low in podocytes according to the present data. Taken together, TMEM16F does not participate in the control of renal filtration under physiological conditions. A subsequent study could examine whether an inflammatory challenge, i.e., induction of glomerulonephritis, unmasks a role of TMEM16F.

## 4. Methods

### 4.1. Cell Culture and Generation of Inducible Cell Lines

Human immortalized podocytes (AB8/13) [19]; kindly provided by Dr. M. Saleem), HEK293 cells (Thermo Scientific, Waltham, MA, USA), and the Retro-X packing cell line GP2-293 were cultured under standard conditions as described earlier [29]. A lentiviral system (pInducer21/10) was used for the generation of stable inducible cell lines [29]. PInducer10 was used for stable inducible knockdown and pInducer21 was employed for inducible overexpression as described before. In brief, short hairpin RNA was designed with the shRNA prediction algorithm provided at http://biodev.extra.cea.fr/DSIR/DSIR.html [37]. In total, six different short hairpin RNA fragments were generated and cloned into the pInducer10 red fluorescent protein (RFP) plasmid as described previously [18]. The resulting pInducer10 (RFP) plasmids encoded short hairpin RNA and a separately transcribed red fluorescent protein (RFP). Expression of short hairpin RNA (sequences as provided previously [18] and RFP was induced by adding 125 ng/mL doxycycline to the medium. Efficient knockdown of the target protein TMEM16F was verified by semiquantitative RT-PCR and Western blotting.

### 4.2. Cell Lysates and Western Blotting

Immunoblotting was carried out as previously indicated [29]. Medium was taken off equally confluent cell culture plates and replaced by 1× Laemmli (4% SDS, 5% 2-mercaptoethanol, 10% glycerol, 0.002% bromophenol blue, 0.0625 M Tris-HCl; pH 6.8). Cell lysates were incubated on a shaking plate for 30 min at room temperature. Afterwards, cell lysates were homogenized via pipetting up and down and then frozen for at least 2 h. After boiling for 3 min, equal volumes of cell lysates were separated on 8–15% SDS-PAGE gels (Biorad, Munich, Germany). Proteins were transferred to a PVDF membrane (Millipore, Darmstadt, Germany) and incubated for 1 h at room temperature in blocking buffer (5% BSA powder dissolved in TBS containing 0.05% Tween-20 (TBS-T)). The lysates were equalized using β-tubulin (Sigma, Munich, Germany; #T2200) as loading controls. TMEM16F (#HPA038958) and anti-FLAG (F3165) were from Sigma. Antibodies against Caspase 3 (#9665), cleaved Capase 3 (Asp175) (#9664), PARP (#9542), cleaved PARP (#5625), p42/44 MAPK (#4695), phospho- p42/44 MAPK (Thr202/Tyr204) (#43769), AKT (#9272), phospho- AKT (Thr308) (#2965), GAPDH (#5174)), and LC3 I/II (#12741) were all from Cell Signaling (Frankfurt, Germany). Horseradish peroxidase-conjugated secondary antibodies were from Dianova (Hamburg, Germany). All primary antibodies were used in a 1:1000 dilution in TBS-T and incubated at 4 °C overnight. After washing three times with TBS-T, the membrane was incubated with horseradish peroxidase-coupled secondary antibodies (Jackson Immunoresearch, Westgrove, PA, USA) diluted 1:5000 (anti mouse) or 1:10000 (anti rabbit) in 5% BSA powder dissolved in TBS-T for 30–60 min at room temperature. Afterwards, the blot was washed three times with TBS-T. Western blots were developed with chemiluminescence detection reagent (Roche, Mannheim, Germany). Signals were collected with X-ray film.

### 4.3. Immunofluorescence

Immunofluorescence was performed as described earlier [29]. In brief, cells were fixed with 4% paraformaldehyde supplemented with 4% sucrose in PBS for 20 min. All steps were performed at room temperature. Samples were washed with PBS and incubated with 50 mM NH4Cl in PBS to quench reactive amino groups. After continued washing, cover slips were permeabilized with PBS containing 0.2% gelatin and 0.2% TritonX-100 (PBS-TG). Then, samples were blocked with 10% goat serum diluted in PBS-TG for 20 min. Immunofluorescence staining was performed by incubating the cover slips for one hour with primary antibodies diluted in PBS-TG containing 2% goat serum. Afterwards, cover slips were washed in PBS-TG and incubated with fluorochrome-conjugated secondary antibodies diluted 1:1000. Nuclei of cells were stained with DAPI (dilution 1:5000). After washing with PBS, cover slips were rinsed in distilled water and cells were mounted in Mowiol. Samples were examined with an Axio Observer Z1 microscope and ApoTome technology (Zeiss, Oberkochen, Germany; objective: EC Plan Neofluar 40×/1.30*Oil DIC M27) using Axio Vision 4.7. The TMEM16F antibody was purchased from Sigma (HPA038958), and antibodies against nephrin were obtained from Invitrogen. For detection of TMEM16F by peroxidase reaction, sections were incubated after exposure to anti-TMEM16F AB in a biotinylated donkey anti-rabbit antibody diluted 1:400 (Santa Cruz Biotechnology, Heidelberg, Germany). After washes, the sections were incubated with di-aminobenzidine. Tissues were counterstained with hematoxylin (Merck, Darmstadt, Germany) before mounting in Depex (SERVA Electrophoresis, Heidelberg, Germany). Nuclei were stained with Hoe33342 (Sigma-Aldrich, Taufkirchen, Germany) and then mounted in DakoCytomation media. Endogenous peroxidase activity was blocked by incubating in 3% H2O2 in PBS.

### 4.4. MTT assay

Determination of cytotoxicity was performed using an MTT assay. The MTT assay was performed as previously described [18]. In brief, cells were grown on translucent flat-bottomed 96-well microtiter plates. On the day of the experiment, cells were provided with 100 µL culture medium. For the assay, 10 µL tetrazolium MTT (3-(4,5-dimethylthiazolyl-2)-2,5-diphenyltetrazolium bromide) were added and cells were incubated at 37 °C for 60 min. During this time, the yellow tetrazolium salt MTT is converted to purple formazan crystals by intracellular reducing equivalents in metabolically active cells. After 3 h incubation, 100 µL lysis buffer (20% sodium dodecyl sulfate, 50% *N*,*N*-dimethylformamide, containing, 0.5% acetic acid (80%), 0.4% 1N HCL) was added to each well and incubated on the shaking plate for 3 h. The optical density was assessed with a fluorescence plate reader (Tecan) at 570 nm.

### 4.5. Generation of Mice with LoxP-Flanked TMEM16F Gene

Conditional knockout of TMEM16A in mice was performed with approval of the federal guidelines for animal welfare. The TMEM16F targeting construct (pTMEM16F_targ.) was designed as follows. The mouse genomic DNA fragment, containing a 4.0 kb right flanking region including exon 8 and intronic sequences was PCR amplified with the oligonucleotides TMEM16F_FlAd2 and TMEM16F_FlAr2 and subcloned. The 1.2 kb left flanking region including intron 6 genomic sequences was PCR amplified using the oligonucleotides TMEM16F_FlBd1 and TMEM16F_FlBr1 and subcloned (compare Table 1 for list of primers). The 0.3 kb exon-7-containing region together with intronic sequences was also PCR amplified with the help of the oligonucleotides TMEM16F_ex7d2 and TMEM16F_ex7r2 and was consequently subcloned. The exon 7 flanking LoxP and the *Hind*III sites were introduced by PCR cloning using the oligonucleotide TMEM16F_ex7r2. All clones were verified by sequencing and assembled into the final construct (Figure 8A–D). The pBluescript-based plasmid backbone combined with the negative selection marker (a thymidine kinase cassette and a diphtheria toxin gene) fused to the left flanking region (not shown). The neomycin cassette flanked by two FRT sites and one LoxP site (positive selection marker) was cloned as an *Eco*RI–*Bam*HI DNA fragment between the left flanking region and the 0.3 kb exon 7 genomic PCR clone. The phCAS9 plasmid, containing cas9 gene and a pgRNA-cloning vector for cloning the gRNA against the *TMEM16F* mouse gene, was received through the Addgene plasmid repository. The cloning of the target sequence AGAATCACTAATTATTAAA was performed according to previous work [38]. The 1.0 kb HR probe (outside of the targeted homology) was PCR amplified from mouse genomic DNA using the oligonucleotides TMEM16F_SoD1 and TMEM16F_SoR1 and subcloned.

CV19 ES cells (passage 13 (129Sv × C57BL/6J)) were grown in HEPES-buffered Dulbecco’s modified Eagle’s medium supplemented with nonessential amino acids, 15% fetal bovine serum (PAA), β-mercaptoethanol, L-glutamine, 1000 U of recombinant leukemia inhibitory factor (MERCK Millipore) per mL, and antibiotics (penicillin (100 U/mL) and streptomycin (100 mg/mL)). For electroporation, 2 × 10^7^ cells were resuspended in 0.8 mL Capecchi buffer (20 mM HEPES (pH 7.4), 137 mM NaCl, 5 mM KCl, 0.7 mM Na_2_HPO_4_, 6 mM dextrose, 0.1 mM β-mercaptoethanol [39]). The pTMEM16F_targ. vector was linearized with *Not*I, and 100 µg of DNA were electroporated together with the CRISPR/cas9 coding plasmids (70 µg DNA of each) at 25 µF^2^ and 400 V in 0.8 mm electroporation cuvettes (Gene Pulser; Bio-Rad, Munich, Germany). Cells after electroporation were kept for 10 min at room temperature and consequently plated onto 10 100-mm diameter culture dishes with a gamma-irradiated monolayer of mouse fibroblast feeder cells. Thirty-two hours later, 350 µg of G418 (Invitrogen) per mL and 0.2 mM 2′-deoxy-2′-fluoro-d-arabinofuranosyl-5-iodouracil (FIAU) (Moravek Biochemicals and Radiochemicals, Brea, CA, USA) were added to the culture medium. The medium was replaced every day, and colonies were picked and analyzed 8 days after plating. Positively targeted ES cell clones were analyzed using the Southern-blot DNA method. Approximately 5–10 µg of the *Hind*III digested genomic DNA samples were fractionated on 0.8% agarose gels and transferred by capillary transport to GeneScreen nylon membranes (NEN DuPont). Membranes were hybridized with a ^32^P-labeled 1.0-kb probe (Figure 8A–D) and washed with 0.5× SSPE (1× SSPE is 0.18 M NaCl, 10 mM NaH_2_PO_4_, and 1 mM EDTA (pH 7.7)) and 0.5% sodium dodecyl sulfate at 65 °C. After the first screening, a correctly targeted event was confirmed on DNAs isolated from positively targeted ES cells and with mouse tail biopsy DNAs using the same *Hind*III and *Eco*RI digestion.

Correctly targeted ES cells (clone H5) were injected into 3.5-day-old B6D2F1 blastocysts. We injected 12 to 14 ES cells into one blastocoele. Injected blastocysts were kept in KSOM medium (5% CO_2_, 37 °C) and afterwards transferred into the uteri of CD-1 foster mice. The mice carried pups to term. Chimeras were identified by their agouti coat color contribution. High percentage male chimaeras were crossed to the C57BL/6J female mice. Heterozygous agouti offsprings (*TMEM16F+/−*) were confirmed by Southern blot analysis (Figure 8E). Mice were kept in specific pathogen-free animal facilities. The subjects were male and female *TMEM16F*-deficient mice. All mouse procedures were performed in compliance with the guidelines for the welfare of experimental animals issued by the Federal Government of Germany. Pups were weaned at 19 to 23 days after birth, and females were kept separately from males. General health checks were performed regularly in order to ensure that any findings were not the result of deteriorating physical conditions of the animals.

### 4.6. Flow Cytometry and LDH Release

PtdSer exposure was assessed using flow cytometry. Cells were removed from the culture dishes with ice-cold PBS. After centrifugation (4 min; 1000× *g*), the supernatant was discarded and Annexin V-FITC (BD Biosciences, #556419) 2.5 µL in 50 µL staining buffer (containing 1% bovine serum albumin in 50 mM 2-(4-(2-hydroxyethyl)-1-piperazinyl)-ethansulfonic acid buffer (HEPES), pH 7.4) was added. After vortexing, the samples were incubated on ice for 20 min. For measurements at the BD Bioscience (FACSCanto I), 450 µL staining buffer containing DAPI 2 µM were added 5 min prior to the measurement. A total of 50,000 cells (FACSCanto) or 10,000 cells (FACSCalibur) was analyzed. Compensation for the channels (DAPI, FITC, RFP/PE) was calculated using the FACSDiva software (FACScanto) or done manually (FACSCalibur). For assessment of lactate dehydrogenase (LDH) release cells, supernatants of treated cultured cells were collected and measured using the CytoTox96^®^ non-radioactive cytotoxicity assay (Promega) at a wavelength of 490 nm. Percentage of LDH release was calculated as 100× (experimental LDH-spontaneous LDH)/(maximum LDH release-spontaneous LDH).

### 4.7. Animal Phenotyping

Conditional TMEM16F knockout mice were bred with the approval of the federal guidelines for animal welfare. Mice homozygous for loxP-flanked TMEM16F were bred with mice expressing a Six2-dependent *cre*-recombinase. When crossbred with Six2Cre mice, TMEM16F is constitutively knocked out. We compared TMEM16FFF × Six2Cre mice with their *cre*-negative littermates. Animals heterozygous for the floxed allele were not included in the study. Six2 is expressed in an early stage of the kidney development, so in this knockout model tubular and glomerular epithelial cells, including podocytes, were targeted (nephron specific knockout) [30]. The newborn mice were examined daily by professionals. The primary endpoint of our observational study was incident proteinuria. Thus, weekly (young mice) or twice a month (older than six months) they were weighted and spot-urine was taken and urine electrophoresis was performed regularly. Urine was mixed with 2× Laemmli-sample buffer, shaken, and heated at 96 °C for 4 min. The urine samples were loaded onto a 10% SDS gel, stained with coomassie brilliant blue R-250 solution (Sigma-Aldrich, Munich, Germany) for 30 min, and finally destained overnight. Animals were sacrificed at the age of 12 months. At this final time point, urine and whole blood was collected. For both, urine and serum, sodium, potassium, and chloride were measured with an ion-sensitive electrode. Calcium, phosphate, creatinine, magnesium, and protein were measured with a photometric assay, and urea was assessed with an enzymatic photometric test. Kidneys were harvested and one kidney was fixed in 4% paraformaldehyde (pH 7.4) for 24 h and then processed for histology. The other kidney was cut into three slices and snap-frozen.

### 4.8. TUNEL Assay

Terminal deoxynucleotidyl transferase dUTP nick end labeling (TUNEL) assays were performed as described earlier [40]. In brief, the DeadEnd Fluorometrie TUNEL system (Promega, Mannheim, Germany) was used according to the manufacturer’s instructions. Immunofluorescence was detected using an Axiovert 200 microscope equipped with ApoTome. Immunofluorescence was analysed quantitatively using Axio-Vision software (Zeiss, Munich, Germany).

### 4.9. Calcium Measurements

Intracellular Ca^2+^ concentrations were assessed in AB8 podocytes using Fura2 fluorescence as described earlier. Cells were seeded on glass cover slips and loaded with 2 μM Fura-2/AM and 0.02% Pluronic F-127 (Invitrogen, Darmstadt, Germany) in ringer solution (mmoL/L: NaCl 145; KH_2_PO_4_ 0,4; K_2_HPO_4_ 1,6; Glucose 5; MgCl_2_ 1; Ca2 ± Gluconat 1,3) for 1 h at room temperature. Fluorescence was detected in cells perfused with Ringer’s solution at 37 °C using an inverted microscope (Axiovert S100, Zeiss, city, Germany) and a high-speed polychromator system (VisiChrome, Puchheim, Germany). Fura-2 was excited at 340/380 nm, and emission was recorded between 470 and 550 nm using a CoolSnap camera (CoolSnap HQ, Visitron, Puchheim, Germany). [Ca^2+^]i was calculated from the 340/380 nm fluorescence ratio after background subtraction. The formula used to calculate [Ca^2+^]i was [Ca^2+^]i = Kd × (R − Rmin)/(Rmax − R) × (Sf2/Sb2), where R is the observed fluorescence ratio. The values Rmax and Rmin (maximum and minimum ratios) and the constant Sf2/Sb2 (fluorescence of free and Ca2 ± bound Fura-2 at 380 nm) were calculated using 1 μM ionomycin (Calbiochem), 5 μM nigericin, 10 μM monensin (Sigma), and 5 mM EGTA to equilibrate intracellular and extracellular Ca^2+^ in intact Fura-2-loaded cells. The dissociation constant for the Fura-2·Ca^2+^ complex was taken as 224 nmoL/L. Control of experiments, imaging acquisition, and data analysis were done with the software package Meta-Fluor (Molecular Devices, San Jose, CA, USA) and Origin (OriginLab Corporation, Northampton, MA, USA).

### 4.10. Patch Clamping

Patch clamp experiments were performed as described earlier [41]. In brief, patch pipettes were filled with a cytosolic-like solution containing in mM: KCl 30, K-gluconate 95, NaH_2_PO_4_ 1.2, Na_2_HPO_4_ 4.8, EGTA 1, Ca-gluconate 0.758, MgCl_2_ 1.03, d-glucose 5, ATP 3, pH 7.2. TMEM16A was activated by high-pipette Ca^2+^ (1 µM). Extracellular Cl^−^ (145 mM) was replaced by gluconate, HCO_3_^−^, or I^−^. Shifts in the reversal potential of current voltage relationships were used to calculate permeability ratios. (Pipette solution 145 mM). Coverslips were mounted in a perfused bath chamber on the stage of an inverted microscope (IM35, Zeiss) and kept at 37 °C. The bath was perfused continuously with Ringer solution at a rate of 8 mL/min. Patch clamp experiments were performed in the fast whole-cell configuration. Patch pipettes had an input resistance of 2–4 MΩ when filled with cytosolic-like solution. Currents were corrected for serial resistance. Access conductances were monitored continuously and were 60–140 nS. Currents (voltage clamp) and voltages (current clamp) were recorded using a patch clamp amplifier (EPC 7, List Medical Electronics, Darmstadt, Germany), the LIH1600 interface and PULSE software (HEKA, Lambrecht, Germany), as well as Chart software (AD Instruments, Spechbach, Germany). Data were stored continuously on a computer hard disc and analyzed using PULSE software. In regular intervals, membrane voltages (*V*c) were clamped from −100 to +100 mV in steps of 20 mV (holding voltage of −100 mV). Current densities were calculated by dividing whole-cell currents by cell capacitance.

## Figures and Tables

**Table 1:** List of oligonucleotides used for generation of TMEM16F-knockout mice.

Genes	Oligonucleotides
*TMEM16F_FlAd2*	GTCTCAAGCGTCTCTTGGAACGCGTATGTTCCCACAGAAGATGGATCATAACTTA
*TMEM16F_FLAr2*	GCTCTAGACGTCTCTGAGAGCGGCCGCTCACCACGGTGCAGCTTCATCCTA
*TMEM16F_FlBd1*	TGTCGACGCACCATGTTATTGGCACTAAGGA
*TMEM16F_FlBr1*	TGGATCCTTAGTGATTCTATAATAAGGGTGATGAT
*TMEM16F_ex7d2*	TGAATTCTTAAATGGCATGTCTGTCCCTTCTGG
*TMEM16F_ex7r2*	TACGCGTATAACTTCGTATAATGTATGCTATACGAAGTTATAAGCTTGTGTGACAGCTGCCCTGCACATAC
*TMEM16F_SoD1*	TTATAGAGCTGATGTCTTCACTTTGGC
*TMEM16F_SoR1*	AGAGTGGAGCTTTACTTTCTGACACA
